# 5-({[(*E*)-Benzyl­idene­amino]­oxy}meth­yl)-1,3,4-thia­diazol-2-amine

**DOI:** 10.1107/S1600536812003893

**Published:** 2012-02-17

**Authors:** Weiyan Yin, Zhi Wang, Zi-Wen Yang

**Affiliations:** a Hubei Biopesticide Engineering Research Center, Hubei Academy of Agricultural Science, Wuhan 430064, People’s Republic of China

## Abstract

In the mol­ecule of the title compound, C_10_H_10_N_4_OS, the configuration about the C=N double bond is *E*. The dihedral angle between the thia­diazole and benzene rings is 81.1 (1)°. In the crystal, mol­ecules are linked by N—H⋯N and C—H⋯O hydrogen bonds to form a two-dimensional network parallel with the *bc* plane.

## Related literature

For the biological activity of thia­diazol compounds, see: Cressier *et al.* (2009 ▶); Ferrari *et al.* (2011 ▶). For a related structure, see: Boechat *et al.* (2006 ▶). For reference structural data, see: Allen *et al.* (1987 ▶).
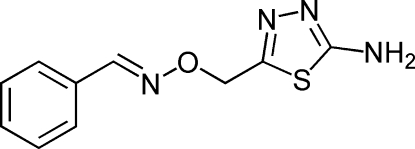


## Experimental

### 

#### Crystal data

C_10_H_10_N_4_OS
*M* *_r_* = 234.28
Monoclinic, 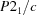

*a* = 14.504 (4) Å
*b* = 9.272 (3) Å
*c* = 8.361 (3) Å
β = 106.75 (1)°
*V* = 1076.7 (6) Å^3^
*Z* = 4
Mo *K*α radiation
μ = 0.28 mm^−1^
*T* = 100 K
0.16 × 0.12 × 0.12 mm

#### Data collection

Bruker SMART APEX CCD diffractometer
Absorption correction: multi-scan (*SADABS*; Bruker, 2000 ▶) *T*_min_ = 0.956, *T*_max_ = 0.967
5840 measured reflections
1808 independent reflections
1707 reflections with *I* > 2σ(*I*)
*R*_int_ = 0.034

#### Refinement

*R*[*F* ^2^ > 2σ(*F* ^2^)] = 0.041
*wR*(*F* ^2^) = 0.110
*S* = 1.09
1808 reflections
145 parameters
H-atom parameters constrained
Δρ_max_ = 0.31 e Å^−3^
Δρ_min_ = −0.61 e Å^−3^

### 

Data collection: *SMART* (Bruker, 2000 ▶); cell refinement: *SAINT* (Bruker, 2000 ▶); data reduction: *SAINT*; program(s) used to solve structure: *SHELXS97* (Sheldrick, 2008 ▶); program(s) used to refine structure: *SHELXL97* (Sheldrick, 2008 ▶); molecular graphics: *SHELXTL* (Sheldrick, 2008 ▶); software used to prepare material for publication: *SHELXTL*.

## Supplementary Material

Crystal structure: contains datablock(s) global, I. DOI: 10.1107/S1600536812003893/fy2038sup1.cif

Structure factors: contains datablock(s) I. DOI: 10.1107/S1600536812003893/fy2038Isup2.hkl

Supplementary material file. DOI: 10.1107/S1600536812003893/fy2038Isup3.cml

Additional supplementary materials:  crystallographic information; 3D view; checkCIF report

## Figures and Tables

**Table 1:** Hydrogen-bond geometry (Å, °)

*D*—H⋯*A*	*D*—H	H⋯*A*	*D*⋯*A*	*D*—H⋯*A*
N4—H4*A*⋯N2^i^	0.88	2.24	3.077 (3)	159
N4—H4*B*⋯N3^ii^	0.88	2.07	2.929 (3)	164
C8—H8*A*⋯O1^iii^	0.99	2.51	3.468 (3)	163

Symmetry codes: (i) 
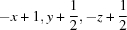
; (ii) 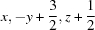; (iii) 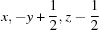.

**Fig. 1.**
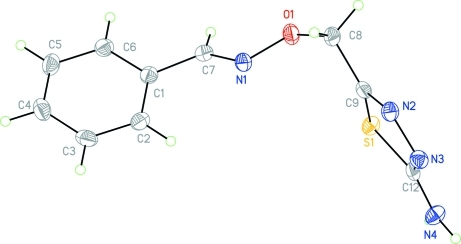
Molecular structure of the title compound, showing 30% probability displacement ellipsoids.

**Fig. 2.**
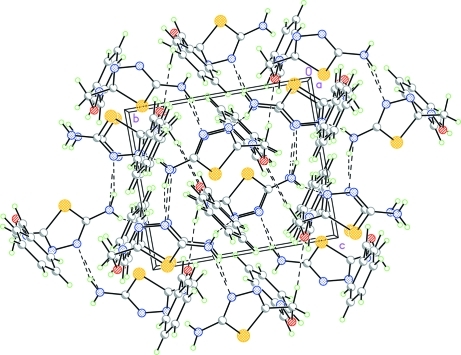
Crystal packing diagram of (I). Hydrogen bonds are shown as dashed lines.

## supplementary crystallographic information

### Comment

The thiadiazol moiety is the constituent of many biologically significant compounds. Thiadiazol derivatives showed diverse biological properties, such as antiparasitic activity (Ferrari *et al.*, 2011), antioxidant properties and radioprotective effects (Cressier *et al.*, 2009). As a part of our study on the synthesis of novel thiadiazol-containing compounds with good biological activities, we report here the crystal structure of the title compound, (I)(Fig. 1).

In the molecule, all bond lengths and angles are normal(Allen *et al.*, 1987). The conformation of the N—H and the C=N bonds in the thiadiazol segment is similar to that observed in other thiadiazol compounds (Boechat *et al.*, 2006). The dihedral angle between the thiadiazol and the benzene rings is 81.1 (1)°. The molecular structure is linked by intermolecular N—H···N and C—H···O hydrogen-bonds to form a two-dimensional network (Table 1, Fig. 2).

### Experimental

To a mixture of aminothiourea (0.43 g, 4.7 mmol) and benzylideneaminooxyacetic acid (0.75 g, 4.3 mmol) phosphorus oxychloride (16.3 mmol) was added dropwise. The reaction mixture was heated at 353 K for 15 min, then cooled to room temperature and water (4.8 mL) was added slowly. After the addtion of water, the reaction mixture was first heated at 383 K for 4 h then cooled to room temperature. The pH of the reaction mixture was then adjusted to 8–9 by addition of 40% aqueous NaOH solution. The crude product was collected by filtration. Single crystals were obtained by evaporation of an ether solution of (I) at room temperature over a period of several days.

### Refinement

The H atoms were placed in calculated positions (C—H = 0.93–0.97Å and N—H = 0.86 Å), and refined as riding with *U*_iso_ (H) = 1.2*U*_eq_(C, N).

### Crystal data

**Table:**

C_10_H_10_N_4_OS	*F*(000) = 488
*M**_r_* = 234.28	*D*_x_ = 1.445 Mg m^−^^3^
Monoclinic, *P*2_1_/*c*	Mo *K*α radiation, λ = 0.71073 Å
Hall symbol: -P 2ybc	Cell parameters from 7867 reflections
*a* = 14.504 (4) Å	θ = 2.2–31.8°
*b* = 9.272 (3) Å	µ = 0.28 mm^−^^1^
*c* = 8.361 (3) Å	*T* = 100 K
β = 106.75 (1)°	Block, colorless
*V* = 1076.7 (6) Å^3^	0.16 × 0.12 × 0.12 mm
*Z* = 4	

### Data collection

**Table:**

Bruker SMART APEX CCD diffractometer	1808 independent reflections
Radiation source: fine-focus sealed tube	1707 reflections with *I* > 2σ(*I*)
Graphite monochromator	*R*_int_ = 0.034
φ and ω scans	θ_max_ = 25.0°, θ_min_ = 1.5°
Absorption correction: multi-scan (*SADABS*; Bruker, 2000)	*h* = −17→15
*T*_min_ = 0.956, *T*_max_ = 0.967	*k* = −11→10
5840 measured reflections	*l* = −9→9

### Refinement

**Table:**

Refinement on *F*^2^	Primary atom site location: structure-invariant direct methods
Least-squares matrix: full	Secondary atom site location: difference Fourier map
*R*[*F*^2^ > 2σ(*F*^2^)] = 0.041	Hydrogen site location: inferred from neighbouring sites
*wR*(*F*^2^) = 0.110	H-atom parameters constrained
*S* = 1.09	*w* = 1/[σ^2^(*F*_o_^2^) + (0.0274*P*)^2^ + 2.2787*P*] where *P* = (*F*_o_^2^ + 2*F*_c_^2^)/3
1808 reflections	(Δ/σ)_max_ = 0.001
145 parameters	Δρ_max_ = 0.31 e Å^−^^3^
0 restraints	Δρ_min_ = −0.61 e Å^−^^3^

### Special details

**Table:**

Geometry. All e.s.d.'s (except the e.s.d. in the dihedral angle between two l.s. planes) are estimated using the full covariance matrix. The cell e.s.d.'s are taken into account individually in the estimation of e.s.d.'s in distances, angles and torsion angles; correlations between e.s.d.'s in cell parameters are only used when they are defined by crystal symmetry. An approximate (isotropic) treatment of cell e.s.d.'s is used for estimating e.s.d.'s involving l.s. planes.
Refinement. Refinement of *F*^2^ against ALL reflections. The weighted *R*-factor *wR* and goodness of fit *S* are based on *F*^2^, conventional *R*-factors *R* are based on *F*, with *F* set to zero for negative *F*^2^. The threshold expression of *F*^2^ > σ(*F*^2^) is used only for calculating *R*-factors(gt) *etc*. and is not relevant to the choice of reflections for refinement. *R*-factors based on *F*^2^ are statistically about twice as large as those based on *F*, and *R*- factors based on ALL data will be even larger.

### Fractional atomic coordinates and isotropic or equivalent isotropic displacement parameters (Å^2^)

**Table:**

	*x*	*y*	*z*	*U*_iso_*/*U*_eq_	
C1	−0.01298 (17)	0.3905 (3)	0.3290 (3)	0.0184 (5)	
C2	−0.03790 (18)	0.4774 (3)	0.1867 (3)	0.0215 (6)	
H2	0.0110	0.5234	0.1502	0.026*	
C3	−0.13385 (19)	0.4967 (3)	0.0985 (4)	0.0252 (6)	
H3	−0.1507	0.5548	0.0007	0.030*	
C4	−0.20545 (19)	0.4310 (3)	0.1531 (4)	0.0264 (6)	
H4	−0.2712	0.4441	0.0925	0.032*	
C5	−0.18121 (18)	0.3468 (3)	0.2954 (4)	0.0253 (6)	
H5	−0.2304	0.3018	0.3320	0.030*	
C6	−0.08548 (18)	0.3276 (3)	0.3849 (3)	0.0215 (6)	
H6	−0.0692	0.2716	0.4843	0.026*	
C7	0.08712 (17)	0.3580 (3)	0.4188 (3)	0.0196 (5)	
H7	0.1040	0.3320	0.5335	0.024*	
C8	0.30958 (17)	0.3047 (3)	0.3626 (3)	0.0209 (6)	
H8A	0.2771	0.2631	0.2517	0.025*	
H8B	0.3607	0.2370	0.4222	0.025*	
C9	0.35421 (16)	0.4469 (3)	0.3404 (3)	0.0178 (5)	
C12	0.42588 (16)	0.6806 (3)	0.3667 (3)	0.0171 (5)	
N1	0.15225 (14)	0.3645 (2)	0.3424 (3)	0.0208 (5)	
N2	0.39251 (14)	0.4743 (2)	0.2222 (3)	0.0196 (5)	
N3	0.43442 (15)	0.6105 (2)	0.2357 (3)	0.0201 (5)	
N4	0.45809 (15)	0.8148 (2)	0.4095 (3)	0.0213 (5)	
H4A	0.4876	0.8624	0.3475	0.026*	
H4B	0.4498	0.8552	0.4995	0.026*	
O1	0.24135 (12)	0.3211 (2)	0.4548 (2)	0.0230 (4)	
S1	0.36427 (4)	0.58523 (7)	0.48329 (8)	0.0189 (2)	

### Atomic displacement parameters (Å^2^)

**Table:**

	*U*^11^	*U*^22^	*U*^33^	*U*^12^	*U*^13^	*U*^23^
C1	0.0195 (12)	0.0160 (12)	0.0206 (14)	−0.0003 (9)	0.0072 (10)	−0.0032 (10)
C2	0.0232 (13)	0.0183 (13)	0.0249 (14)	−0.0024 (10)	0.0100 (11)	−0.0010 (10)
C3	0.0291 (14)	0.0167 (13)	0.0267 (15)	0.0039 (10)	0.0029 (11)	0.0012 (11)
C4	0.0184 (12)	0.0258 (14)	0.0328 (16)	0.0029 (10)	0.0040 (11)	−0.0073 (12)
C5	0.0193 (12)	0.0270 (14)	0.0325 (16)	−0.0021 (11)	0.0122 (11)	−0.0026 (12)
C6	0.0224 (12)	0.0194 (13)	0.0243 (14)	0.0006 (10)	0.0095 (11)	0.0000 (10)
C7	0.0206 (12)	0.0162 (12)	0.0222 (14)	−0.0008 (10)	0.0064 (10)	0.0007 (10)
C8	0.0168 (11)	0.0216 (13)	0.0260 (15)	0.0008 (10)	0.0090 (10)	−0.0007 (10)
C9	0.0144 (11)	0.0186 (12)	0.0202 (13)	0.0033 (9)	0.0049 (10)	0.0010 (10)
C12	0.0119 (10)	0.0237 (13)	0.0163 (13)	0.0037 (9)	0.0051 (9)	0.0027 (10)
N1	0.0165 (10)	0.0211 (11)	0.0218 (12)	−0.0007 (8)	0.0007 (9)	0.0007 (9)
N2	0.0180 (10)	0.0227 (11)	0.0190 (11)	0.0005 (8)	0.0067 (8)	−0.0010 (9)
N3	0.0200 (10)	0.0203 (11)	0.0212 (12)	0.0026 (8)	0.0079 (9)	0.0001 (9)
N4	0.0257 (11)	0.0207 (11)	0.0210 (12)	−0.0044 (9)	0.0122 (9)	−0.0029 (9)
O1	0.0157 (8)	0.0283 (10)	0.0239 (10)	0.0009 (7)	0.0038 (7)	0.0023 (8)
S1	0.0204 (3)	0.0219 (3)	0.0177 (4)	−0.0020 (2)	0.0106 (2)	−0.0010 (2)

### Geometric parameters (Å, º)

**Table:**

C1—C2	1.396 (4)	C8—O1	1.426 (3)
C1—C6	1.396 (4)	C8—C9	1.503 (3)
C1—C7	1.461 (3)	C8—H8A	0.9900
C2—C3	1.386 (4)	C8—H8B	0.9900
C2—H2	0.9500	C9—N2	1.291 (3)
C3—C4	1.390 (4)	C9—S1	1.730 (3)
C3—H3	0.9500	C12—N3	1.309 (3)
C4—C5	1.381 (4)	C12—N4	1.341 (3)
C4—H4	0.9500	C12—S1	1.742 (2)
C5—C6	1.386 (4)	N1—O1	1.419 (3)
C5—H5	0.9500	N2—N3	1.392 (3)
C6—H6	0.9500	N4—H4A	0.8800
C7—N1	1.285 (3)	N4—H4B	0.8800
C7—H7	0.9500		
			
C2—C1—C6	119.5 (2)	O1—C8—C9	111.4 (2)
C2—C1—C7	122.2 (2)	O1—C8—H8A	109.4
C6—C1—C7	118.3 (2)	C9—C8—H8A	109.4
C3—C2—C1	120.1 (2)	O1—C8—H8B	109.4
C3—C2—H2	119.9	C9—C8—H8B	109.4
C1—C2—H2	119.9	H8A—C8—H8B	108.0
C2—C3—C4	120.0 (3)	N2—C9—C8	124.3 (2)
C2—C3—H3	120.0	N2—C9—S1	114.39 (19)
C4—C3—H3	120.0	C8—C9—S1	121.23 (19)
C5—C4—C3	120.1 (2)	N3—C12—N4	125.0 (2)
C5—C4—H4	119.9	N3—C12—S1	113.79 (19)
C3—C4—H4	119.9	N4—C12—S1	121.15 (19)
C4—C5—C6	120.3 (2)	C7—N1—O1	108.5 (2)
C4—C5—H5	119.9	C9—N2—N3	113.0 (2)
C6—C5—H5	119.9	C12—N3—N2	112.0 (2)
C5—C6—C1	120.0 (3)	C12—N4—H4A	120.0
C5—C6—H6	120.0	C12—N4—H4B	120.0
C1—C6—H6	120.0	H4A—N4—H4B	120.0
N1—C7—C1	119.9 (2)	N1—O1—C8	108.28 (19)
N1—C7—H7	120.0	C9—S1—C12	86.84 (12)
C1—C7—H7	120.0		
			
C6—C1—C2—C3	−2.3 (4)	C1—C7—N1—O1	−177.0 (2)
C7—C1—C2—C3	175.2 (2)	C8—C9—N2—N3	−176.0 (2)
C1—C2—C3—C4	0.9 (4)	S1—C9—N2—N3	0.3 (3)
C2—C3—C4—C5	0.1 (4)	N4—C12—N3—N2	−178.9 (2)
C3—C4—C5—C6	0.2 (4)	S1—C12—N3—N2	−0.6 (3)
C4—C5—C6—C1	−1.6 (4)	C9—N2—N3—C12	0.2 (3)
C2—C1—C6—C5	2.6 (4)	C7—N1—O1—C8	170.3 (2)
C7—C1—C6—C5	−175.0 (2)	C9—C8—O1—N1	83.4 (2)
C2—C1—C7—N1	−24.1 (4)	N2—C9—S1—C12	−0.50 (19)
C6—C1—C7—N1	153.4 (2)	C8—C9—S1—C12	175.9 (2)
O1—C8—C9—N2	−156.5 (2)	N3—C12—S1—C9	0.63 (19)
O1—C8—C9—S1	27.5 (3)	N4—C12—S1—C9	179.0 (2)

### Hydrogen-bond geometry (Å, º)

**Table:**

*D*—H···*A*	*D*—H	H···*A*	*D*···*A*	*D*—H···*A*
N4—H4*A*···N2^i^	0.88	2.24	3.077 (3)	159
N4—H4*B*···N3^ii^	0.88	2.07	2.929 (3)	164
C8—H8*A*···O1^iii^	0.99	2.51	3.468 (3)	163

Symmetry codes: (i) −*x*+1, *y*+1/2, −*z*+1/2; (ii) *x*, −*y*+3/2, *z*+1/2; (iii) *x*, −*y*+1/2, *z*−1/2.
